# Editorial: The Commoditization of Medical Physics

**DOI:** 10.1120/jacmp.v13i3.3942

**Published:** 2012-05-10

One of my favorite authors is Thomas Friedman, a columnist for *The New York Times*, who writes, among other topics, about the globalization of our $21^{\text{st}}$ century economy. His most recent book, *That Used to be Us* (That used to be us: how America fell behind in the world it invented and how we can come back. New York, NY: Farrar, Straus and Giroux; 2011), which he co‐authored with Michael Mandelbaum, addresses what the authors perceive as the decline of inventiveness in the United States. In their book, the authors describe four types of workers. The first type is called “creative creators.” These are individuals who do nonroutine work in a nonroutine way. These individuals are the top professionals: the best lawyers, accountants, physicians, scientists, teachers, and medical physicists. The second category is the “routine creators.” These are individuals who do nonroutine work, but in a routine way: the average lawyers, accountants, physicians, scientists, teachers, and medical physicists. The third category is the set of individuals called “creative servers.” These are the lesser‐skilled individuals who, nonetheless, do their jobs in inspired ways. For example, the baker who has a special cake recipe or the nursing aide who displays inspired bedside skills in the nursing home. The final category is the “routine servers,” individuals who do routine work in routine way, offering nothing extra.

As medical physicists, we like to place ourselves in the category of “creators,” but, based on the classifications described by Friedman and Mandelbaum, we need to ask ourselves whether we are creative creators or routine creators. Following the reasoning of Friedman and Mandelbaum, just because our work is not routine, that does not immunize us from such job‐killing actions as outsourcing or automation. If you are a routine creator, your work becomes a commodity, a product that can be sold to the lowest bidder. Ask yourself the following question: What do you bring to your work as a medical physicist that is unique? Even though you possess expertise that is relatively scarce, how can you be sure that your work will not eventually be replaced by someone who can do it faster, more efficiently, at less cost? Worse yet, from your point of view, can you eventually be replaced by a machine? As Friedman recently wrote, “Average is officially over.”^(^ ^1^ ^)^

To illustrate this point, look, for example, at some of the routine tasks that have been performed by clinical medical physicists. One of these tasks is the weekly checking of radiation oncology treatment charts. Years ago, when treatment times and doses were filled in manually, we would regularly find errors in the charts, primarily in the addition of doses. Continual review of these charts was necessary to ensure that the patient received the correct number of treatments and the prescription goals were met. After several years as a medical physicist checking addition on a weekly basis, I moved to another institution that had a practice of “dosing out” charts, in which the dosimetrist would pencil in what the accumulated doses should be in advance, and flag points at which treatment needed to be changed or terminated. This simple action reduced errors and made the manual checking of arithmetic much more routine. Then we moved to electronic charts, in which all the addition and dose recording was done automatically and accurately. The next logical step is to develop “intelligent charts,” containing software that would compare the numbers in a patient's chart to a database of patient charts, flagging only those charts for which there might be some deviation from a site‐specific, prescription‐specific standard. Such an approach is likely to be more efficient, more reliable, and ultimately more cost effective, than human checking. What happens to the average medical physicist then?

Another example lies in the routine machine output and energy checks that are performed regularly on linear accelerators. Linear accelerators are becoming extremely stable and reliable, so that vendors have been suggesting that regular checking may not be necessary. What if we add an external monitor that would continuously measure the output and beam energy while minimally perturbing the beam, warning us only if output and energy varied by more than a specified amount? Would we feel comfortable abandoning routine checks for continuous monitoring? This method would be more reliable and less costly than having a physicist perform these checks on a regular basis.

Many medical physicists follow one or more of the medical physics list servers. Recently, a poster on the MedPhyUSA list server wrote the following:

“I can think of nothing more luxurious than the mundane repetition of a clinical physics job and its regular, thick paychecks.”

A reply came back, saying:

“… if you find your job to be mundane repetition, you are probably in the wrong field, and are depriving your patients and your facility of the true effort that they deserve. I have always found that I could always do more for my patients, whether that be directly for the individual, researching new methods, suggesting departmental improvements, knowing the limitations of the equipment and of software, etc.”

In the first case, the individual is a routine creator, for whom medical physics is a commodity, whereas the responder is a creative creator, who has decommoditized medical physics.

Medical physicists need to be continually ahead of technology, ensuring that new technologies are introduced into the clinic in a manner that is safe and effective. Medical physicists need to develop new, effective ways of performing clinical responsibilities, doing our nonroutine work in creative, nonroutine ways, adding value to the work we do, and decommoditizing medical physics.

And, of course, when you have developed a new and effective way of performing your clinical responsibilities, please share your newfound clinical expertise with your colleagues by submitting your work for publication in the JACMP.

George Starkschall, PhD

Editor‐in‐Chief

May 10, 2012

## Footnotes

1. Available from: http://www.nytimes.com/2012/01/25/opinion/friedman‐average‐is‐over.html?_r=1&hp

